# Development of an objective gene expression panel as an alternative to self-reported symptom scores in human influenza challenge trials

**DOI:** 10.1186/s12967-017-1235-3

**Published:** 2017-06-08

**Authors:** Julius Muller, Eneida Parizotto, Richard Antrobus, James Francis, Campbell Bunce, Amanda Stranks, Marshall Nichols, Micah McClain, Adrian V. S. Hill, Adaikalavan Ramasamy, Sarah C. Gilbert

**Affiliations:** 1 The Jenner Institute, University of Oxford, Old Road Campus Research Building, Oxford, OX3 7DQ UK; 2 Immune Targeting Systems Ltd (now AltImmune Ltd), 2 Royal College Street, London, NW1 0NH UK; 3 Center for Applied Genomics and Precision Medicine, Duke University Medical Centre, Durham, NC 27708 USA

**Keywords:** Influenza, Challenge trial, Symptom scores, Biomarker, Transcriptomics

## Abstract

**Background:**

Influenza challenge trials are important for vaccine efficacy testing. Currently, disease severity is determined by self-reported scores to a list of symptoms which can be highly subjective. A more objective measure would allow for improved data analysis.

**Methods:**

Twenty-one volunteers participated in an influenza challenge trial. We calculated the daily sum of scores (DSS) for a list of 16 influenza symptoms. Whole blood collected at baseline and 24, 48, 72 and 96 h post challenge was profiled on Illumina HT12v4 microarrays. Changes in gene expression most strongly correlated with DSS were selected to train a Random Forest model and tested on two independent test sets consisting of 41 individuals profiled on a different microarray platform and 33 volunteers assayed by qRT-PCR.

**Results:**

1456 probes are significantly associated with DSS at 1% false discovery rate. We selected 19 genes with the largest fold change to train a random forest model. We observed good concordance between predicted and actual scores in the first test set (r = 0.57; RMSE = −16.1%) with the greatest agreement achieved on samples collected approximately 72 h post challenge. Therefore, we assayed samples collected at baseline and 72 h post challenge in the second test set by qRT-PCR and observed good concordance (r = 0.81; RMSE = −36.1%).

**Conclusions:**

We developed a 19-gene qRT-PCR panel to predict DSS, validated on two independent datasets. A transcriptomics based panel could provide a more objective measure of symptom scoring in future influenza challenge studies.

*Trial registration* Samples were obtained from a clinical trial with the ClinicalTrials.gov Identifier: NCT02014870, first registered on December 5, 2013

**Electronic supplementary material:**

The online version of this article (doi:10.1186/s12967-017-1235-3) contains supplementary material, which is available to authorized users.

## Background

Seasonal influenza vaccination is widely used but has low effectiveness despite annual revaccination [1]. When the main circulating influenza strains have drifted away from those included in that season’s vaccine, effectiveness can be very low [2]. Live attenuated influenza virus vaccines have become the preferred vaccine for use in children due to the ability to induce T cell responses to the influenza virus as well as humoral responses to the external glycoproteins, but recent findings of low vaccine effectiveness in the US [3, 4] have resulted in the recommendation to use inactivated vaccines only in the US this season. Seasonal influenza vaccines cannot provide protection against influenza pandemics caused by novel subtypes, and much research effort has been directed towards producing a ‘universal’ influenza A vaccine that will protect against any subtype of influenza A. This has resulted in a number of different approaches to vaccination against influenza which have entered early phase clinical development [5–8]. Seasonal influenza vaccines are licensed based on their ability to induce a haemagglutination inhibition (HI) titre of 1:40, but for novel vaccines that are designed to act through alternative immune mechanisms it will be necessary to demonstrate efficacy of the vaccine in preventing disease caused by influenza viruses, in humans.

Influenza challenge trials provide one means of testing vaccine efficacy, and whilst they are not inexpensive to conduct, are considerably less costly than phase III clinical trials involving many tens of thousands of subjects, and may be conducted more rapidly to give a preliminary assessment of the protective efficacy of a novel vaccine against seasonal influenza viruses. However, conducting efficacy studies in a cohort of humans in a quarantine unit presents challenges in the collection of sufficient objective data points for analysis. Shedding of virus collected in nasal swabs or washes can only be measured once per day, up to 7 days after influenza challenge. Temperature measurements are taken at least twice per day but fevers are rare in healthy young subjects after influenza virus challenge. The majority of the data that is collected to determine the severity of illness consists of self-reported symptom scores. Subjects record whether each of a list of possible influenza symptoms is absent, mild, moderate or severe in a twice daily questionnaire. Symptom scoring may be influenced by many factors such as the mood of the volunteer, the time elapsed since waking, or drinking, or the perception that the volunteer should be feeling better in the later part of the quarantine period.

Earlier time course transcriptomics analysis of data from human influenza challenge trials [9–13] have primarily focused on identifying genes and transcriptional dynamics that are different between clinically symptomatic from asymptomatic individuals at various time points post challenge. The definition for symptomatic and asymptomatic varies between studies but is typically based on total symptom score in the first few days and may include a secondary restriction on virus shedding status.

To our knowledge, no studies have attempted to predict the per individual symptom score using gene expression data using a minimal set of biomarkers. Such a panel would be a more objective measure of symptom scoring in future influenza challenge trials and thus improve comparability between challenge studies, especially those testing different candidate vaccines.

## Methods

### Influenza challenge study (discovery cohort)

Samples were obtained from a clinical trial (ClinicalTrials.gov Identifier: NCT02014870) conducted to determine the appropriate dose level of live, wild-type A/California/2009 H1N1 virus stock for future influenza challenge studies [14]. Healthy volunteers aged 18–45 with no detectable HAI titre to the challenge strain underwent intranasal administration of virus whilst housed in a quarantine unit, and were monitored for symptoms of influenza disease and virus shedding. Volunteers in a semi-recumbent position were intranasally challenged with 0.5 mL (0.25 mL in each nostril) of either 1:100 or 1:10 dilution of the neat virus (concentration ~7.0 × 10^7^ TCID_50_/mL).

Volunteers were quarantined for 9 days after challenge and self-reported twice daily on 16 signs and symptoms of influenza. Symptoms were recorded on a modified Jackson score 0–3: not noticeable, just noticeable, bothersome but can still do daily or bothersome and cannot do daily activity. Nasal swabs were taken daily to determine the live virus shedding load. In consenting subjects, blood samples were collected in PAXgene^®^ tubes before the challenge and 24, 48, 72 and 96 h post challenge for transcriptomics analysis.

### RNA extraction and quality control

Whole blood was collected in PAXgene Blood RNA tubes (PreAnalytiX) and processed according to the manufacturer’s protocol. RNA quantity and quality were assessed using a NanoDrop spectrophotometer and Agilent’s 2100 Bioanalyzer.

### High throughput qPCR

Real-time quantitative PCR was performed in a Fluidigm system consisting of a BioMark HD instrument, IFC HX controller and 96 × 96 dynamic array, as described in the manufacturer’s user guide PN 68000088 K1 [15] (“Real-Time PCR Analysis”, appendixes A and D) and document PN 100-2638 D1 [16] (“Gene Expression with the 96.96 IFC Using Fast TaqMan Assays”). Appendix A in guide PN 68000088 K1 was used for the preparation of cDNA through reverse transcription and appendix D contains the protocol for gene expression analysis using TaqMan assays, including a preamplification step. Raw RNA samples were normalised to 10 ng/μL for the cDNA synthesis and the expression assay involved three technical replicates.

### Gene expression microarrays

Blood samples in PAXgene^®^ tubes were thawed over 2 h at room temperature and total intracellular RNA was extracted using the Blood RNA Kit (Qiagen) according to the manufacturer’s instructions. The purity and quantity of the isolated total RNA was assessed using an Agilent Bioanalyser prior to storage at −20 °C until required. Globin mRNA was subsequently depleted using the GLOBINclear Kit (Ambion). Depleted RNA was then amplified and biotin-labelled using the TotalPrep RNA Amplification Kit (Illumina) and RNA quality assessed using Agilent’s 2100 Bioanalyzer. This was purified and assessed using the Agilent bioanalyser. Biotinylated cRNA was hybridised to Illumina Human HT-12 v4 Expression Beadchips according to the manufacturer’s instructions. Beadchips were scanned with an Illumina iScan machine, and data extracted using the Illumina’s GenomeStudio 2011 software.

### Microarray analysis

Raw probe level summary were exported from GenomeStudio 2011 and imported into R using the beadarray package [17]. Probes were background corrected using negative control probes followed by quantile normalization using the neqc command [18]. The analysis was restricted to probes with a detection p value <0.01 in at least 10% of the samples and probes matching to the transcript definition of the following databases (in descending importance) with at most two mismatches, no insertions and a minimum mapping length of 40 bases: GENCODE version 23, RefSeq (refMrna.fa) and GenBank (mrna.fa) downloaded in August 2015 from http://hgdownload.cse.ucsc.edu/goldenPath/hg38/bigZips/. A linear model was fitted using limma [19] to determine differential expression adjusted for gender, age, challenge dose and batch effects. We used the duplicate correlation option [20] to account for intra-patient correlations and weighted the arrays by their quality scores [21]. Nominal p values were corrected for multiple hypothesis testing using the Benjamini–Hochberg procedure [22].

### High-throughput qPCR analysis

We performed TaqMan^®^ Gene Expression assays to determine gene expression in the whole-blood RNA isolated from selected participants at baseline and the time of maximal symptoms. A panel of 29 primers selected for symptom scoring and three control primers were measured in triplicates on a 96 × 96 Fluidigm plate. One sample and one primer did not meet the quality criteria and were removed from the analysis. The raw Ct values were then imported into R and normalized to the three endogenous control genes (RPL30, GAPDH, and PPIA) into log transformed deltaCt values using the HTqPCR package in R [23]. The limmaCtData command was used to extract fold changes to DSS.

### Regression analysis

For the prediction of DSS on external test sets, microarray intensities and deltaCt values were used to train a random forest model using the R package caret [24]. The microarray data for training were the residuals after removing the effect of gender, age, challenge dose and scan dates. Three technical replicates were averaged. For genes with multiple probes, we retain only the highest expressing probe. To estimate the training performance, training samples were randomly sampled and out of bag RMSE (root mean squared error) estimates were used to select the optimal tuning parameter mtry. The final hyper parameters chosen for the microarray data were mtry = 2, ntree = 500, sampsize = 105 and for the qRT-PCR panel mtry = 19, ntree = 500, sampsize = 33. Test sample DSS was then predicted using the trained model and prediction performance was evaluated using the percent change in RMSE relative to a best guess model predicting the overall mean. To select a minimal set of predictive features, the VSURF package was used with default settings in R [25].

### External validation dataset

We used two external datasets from Influenza A challenge trials to validate our findings. The first is from Woods et al. [13], in which 24 volunteers were experimentally infected with H1N1 (A/Brisbane/59/2007) and 17 volunteers with H3N2 (A/Wisconsin/67/2005). Blood was collected approximately every 8 h for transcriptomics analysis but volunteers only self-reported twice daily. We downloaded the RMA processed data from Gene Expression Omnibus website (GSE5428) and only used microarray data (~70%) that was collected within 3 h of symptom reporting. The self-reported scores were to ten signs and symptoms which was a subset used to define DSS in the discovery cohort. The data was generated using Affymetrix Human Genome U133A 2.0 Array. We selected the highest expressing Affymetrix probe for genes with multiple probes and matched to the Illumina probes. The second dataset was used to confirm results obtained with the Fluidigm panel and contains samples from placebo-vaccinated subjects in an independent H1N1 challenge trial (ClinicalTrials.gov Identifier: NCT02071329) conducted by Immune Targeting Systems (ITS) Ltd using the same challenge strain and protocols as the discovery cohort. Blood samples were collected from 30 adult volunteers before challenge and 72 h post challenge in PAXgene^®^ tubes, and RNA was extracted before profiling gene expression using the Fluidigm panel as described above.

## Results

### Challenge study outcome

**Fig. 1:**
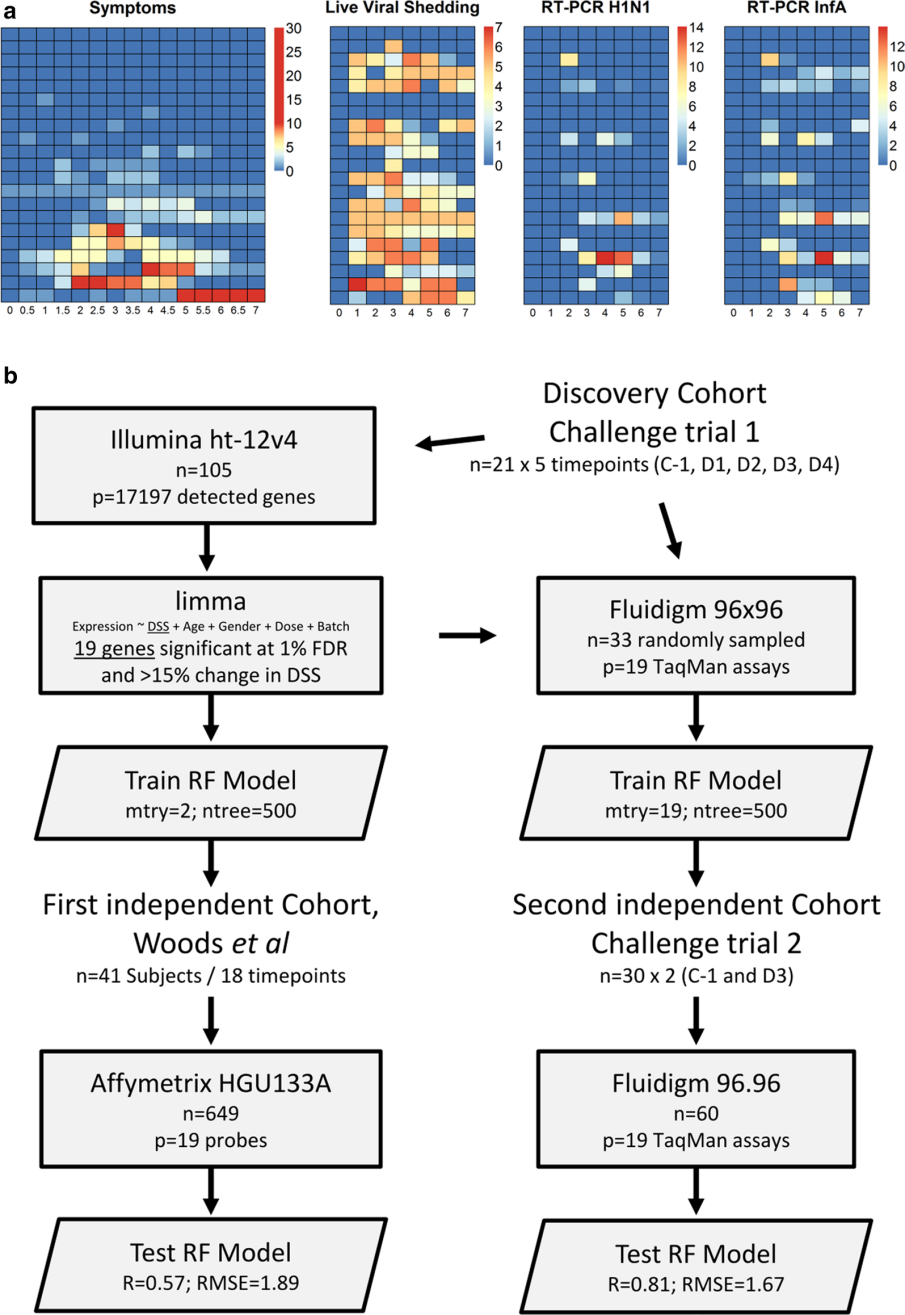
**a** Heatmap of summed symptom scores, live viral shedding assay, RT-PCR Influenza A and RT-PCR H1N1. Each *row* represents one subject and each *column* one time point in days post challenge. **b** Schematic overview of the three cohorts used and the supervised learning approach used for the regression analysis

A human influenza challenge trial was conducted where 21 healthy adult volunteers were intranasally challenged with live influenza A (wild-type A/California/2009 H1N1) virus. Each patient was quarantined for 9 days after challenge and reported twice daily on 16 signs and symptoms of influenza. Symptoms were recorded on a 0–3 scale and peak symptoms occurred at 72–96 h post challenge (Fig. 1a; Additional file 1: Figure S1A, Additional file 2: Table S1, Additional file 3: Table S2).

Nine volunteers reported no symptoms or only one mild symptom within a week of challenge but six of these had some detectable amounts of live virus shedding. The remaining 12 volunteers reported more than one mild symptom and detectable amounts of live virus shedding. Volunteer 204 reported a mild nasal congestion before and throughout the challenge which we set to zero as we felt it was unrelated to the challenge trial.

### Whole blood transcriptome microarray analysis

Volunteers self-reported their symptoms twice daily on a list of 16 conditions using the modified Jackson scoring on a scale of 0–3 per condition. We calculated the daily sum of scores (DSS) using only the morning report, which coincided very closely with phlebotomy time for samples taken for microarray, by summing up the scores over the 16 conditions. The DSS values ranged between 0 and 23. We identified 1456 probes significantly associated with DSS at FDR <1%. To investigate the consistency of the panel of symptoms, we correlated changes in expression of all genes for each individual symptom to the changes in expression to DSS (Additional file 1: Figure S1B). The correlation was high and ranged from 0.72 to 0.96 and fever, fatigue and nasal congestion were the highest correlated symptoms. It is interesting to note that we obtain a very similar output if we had chosen to analyse the fold increase in live virus shedding (Additional file 4: Figure S2).

### DSS biomarker selection and validation

**Fig. 2:**
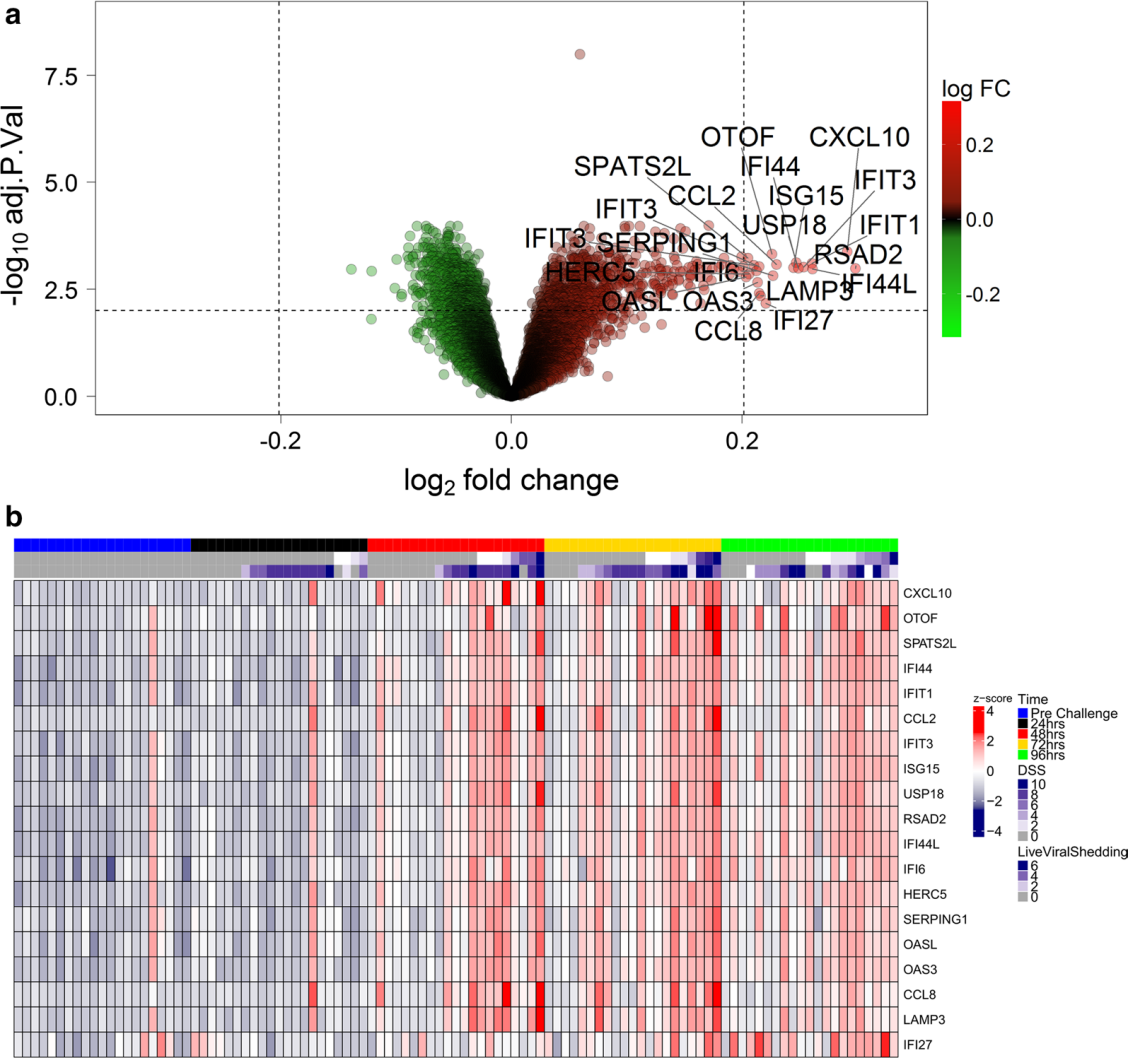
**a** Volcano plot showing all 21 probes (19 genes) associated to significant changes in DSS. The threshold was set to 15% change in DSS per unit in gene expression and a FDR of 1%. **b** Heatmap of scaled expression values of the 19 genes significantly associated to changes in DSS

In order to identify the most predictive genes for DSS, we selected a stringent cut off of 1% FDR and at least 15% unit increase in DSS per log2 unit increase in expression. This resulted in a list of 21 probes mapping to 19 candidate biomarkers (Fig. 2; Additional file 5: Figure S3). Eleven of the 19 markers are associated to the GO category “response to virus” (CCL8, HERC5, IFIT1, IFIT3, ISG15, OAS3, OASL, RSAD2, CXCL10, IFI44 and IFI44L), 5 of the remaining 8 are associated to the GO category “innate immune system” (CCL2, IFI27, IFI6, SERPING1 and USP18) and LAMP3 is associated to the GO category “adaptive immune response”. Also, 14 of the 19 genes were previously included in a panviral gene signature predicting viral shedding [10]. The gene with the highest predictive importance, CCL2, was ranked as the most predictive gene for laboratory confirmed influenza in [11] and in the same study OTOF and SPATS2L have been among the 100 most upregulated genes.

**Fig. 3:**
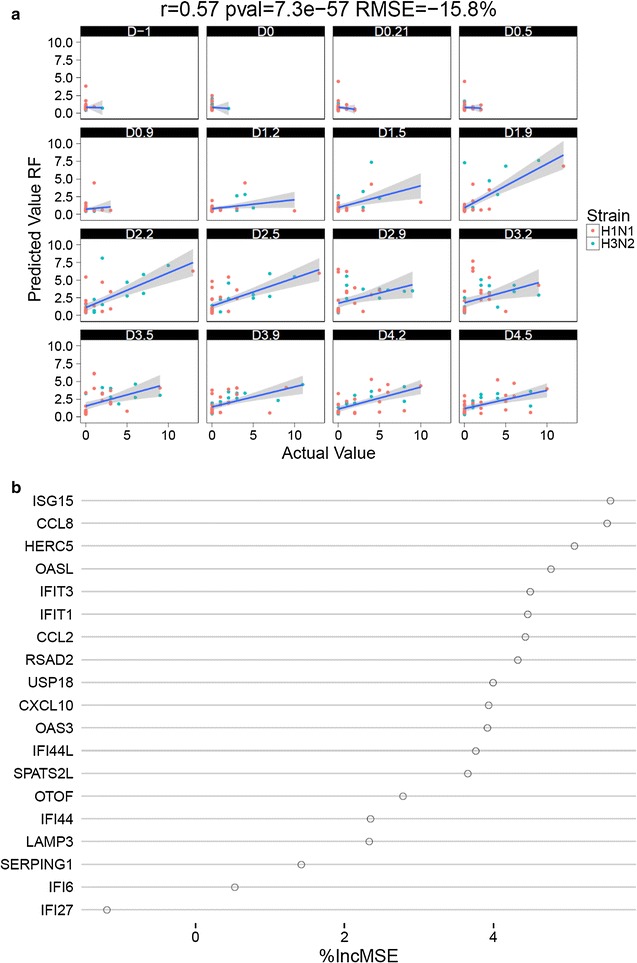
Intensities from Illumina ht-12 arrays of the 19 gene signature were used to train a random forest model. The model was used to predict symptom scores in an independent test set. **a** Actual scores were plotted against the predicted values at each day. RMSE decrease and correlation values refer to the overall model fit. **b** Importance of individual markers within the panel of 19 primers. X-axis shows the increase in RMSE if the respective gene is left out of the model

We next sought to test the predictive performance of this signature using an independent dataset. First we trained a random forest model using expression levels of the 19 genes in our dataset and predicted DSS in the first independent dataset [13]. Despite differences in microarray platforms, challenge virus strains and list of reported symptoms, we observed a reduction of 15.7% in root mean squared error (RMSE) using predicted DSS compared to a best guess prediction assuming overall mean DSS. Predicted values correlated positively and significant with observed DSS (r = 0.57; p < 7e−57) and prediction accuracies were consistently high between day 1.5 until day 4.5 (Fig. 3).

To further elucidate which time point in the discovery cohort is most predictive, we partitioned the expression of both, training and test data, into the individual time points and predicted DSS across all partitions (Additional file 6: Figure S4). The most increase in accuracy was clearly observed, when training on samples from day 2 to day 4 and predicting on testing samples from day 1.5 to day 4.2 with an average decrease of 16% in RMSE (range −4 to −30%).

### Biomarker panel development

To translate these findings into a small scale qRT-PCR based assay which can be used for symptom prediction in future influenza challenge trials, we selected 19 commercially available primers (Additional file 7: Table S3) for the BioMark HD multiplex microfluidic instrument (Fluidigm, CA, USA). We randomly selected 33 samples from our discovery cohort to train the random forest model. The technical reproducibility between expression level changes associated to DSS in microarrays and Fluidigm assays was very high (R^2^ = 0.75; Additional file 8: Figure S5). Samples from the second independent dataset were also profiled on the Fluidigm using the same primers.

### Validation of biomarker panel and further optimization

**Fig. 4:**
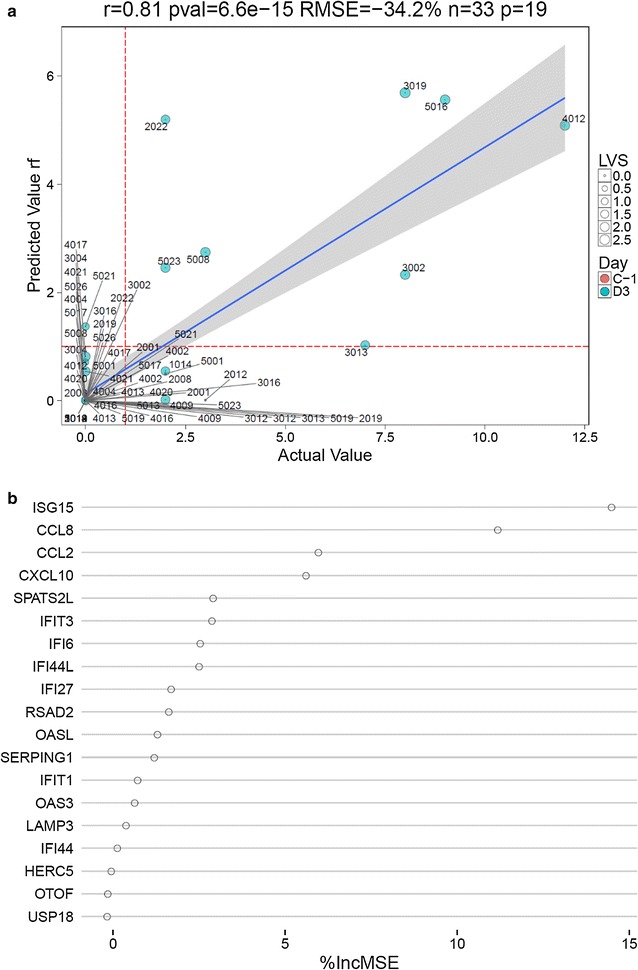
**a** The predicted DSS from the qRT-PCR based assay using the deltaCt values from the 19 gene signature as a test dataset were plotted against the observed DSS. **b** The importance of individual markers within the panel of 19 primers is shown in decreasing importance. The x-axis shows the increase in RMSE if the respective gene is left out of the model. *LVS* live viral shedding assay

A Random Forest model was then trained using delta-Ct values of the 19 genes in our cohort to predict DSS in the second independent cohort. We observed a strong reduction in RMSE of 34% compared to a best guess prediction assuming the overall mean DSS. The correlation between predicted and observed scores was also strong and significant (r = 0.81, p < 6e−15) (Fig. 4).

To evaluate whether the set of 19 genes can be further reduced to an even smaller set of biomarkers, we applied the feature selection method VSURV [25, 26]. This method eliminates redundant and less informative features recursively using a set of three rules with increasing stringency. After the third selection step only two genes, ISG15 and CXCL10, were left. Intriguingly, the RMSE reduction was still substantial with 26% and correlation between predicted and observed scores was strong and significant (r = 0.73, p < 7e−11; Additional file 9: Figure S6A).

Conversely, we tried to improve the model fit by adding more features. Since viral shedding and reported symptom scores are highly correlated, we added an additional set of ten genes highly correlated with the live viral shedding assay to our set of 19 biomarkers and trained a random forest model. Predictive performance did not improve significantly but remained high, comparable to the original signature (RMSE = −34.9%, r = 0.8, p < 2e−14; Additional file 10: Figure S7).

To test whether our signature can be used to accurately predict categorized symptom scores and to compare prediction performance to previous studies, we categorized patients into symptomatic and asymptomatic depending on presence of any DSS at each time point. Prediction accuracy was very high (AUC 0.95; Additional file 11: Figure S8) and 3/13 symptomatic samples were misclassified as asymptomatic and conversely 1/47 asymptomatic was misclassified as symptomatic.

We also tested the reduced signature of the two genes ISG15 and CXCL10 using the linear regression method Partial Least Squares to define easier to interpret decision boundaries (Additional file 9: Figure S6B). Prediction of symptoms classes was less accurate but still high (AUC 0.91; Additional file 9: Figure S6C).

## Discussion

This study was designed to develop a precise, objective and cost effective small scale assay to act as a surrogate for symptom scoring in influenza challenge trials. We challenged 21 volunteers with wild-type A/California/2009 H1N1 virus and 15 out of 21 volunteers or 71% reported at least one mild symptom. This is consistent with the previously reported proportion of H1N1 infected volunteers who develop clinical illness after experimental influenza virus infection of 69% [27]. We generated a whole transcriptome dataset on all 21 volunteers and correlated the longitudinal transcriptomics data with the self-reported symptoms. The most significant differentially expressed genes to DSS were then used to establish a qRT-PCR based Fludigm assay using commercially available primers.

In contrast to previous studies which try to distinguish symptomatic and asymptomatic individuals [9–11], we established a set of biomarkers optimised to detect and differentiate between different levels of symptoms. We showed that the continuous range of predicted symptom scores can be optionally categorized. Importantly, no baseline sample was misclassified when samples were categorized into symptomatic and asymptomatic in our qRT-PCR training and test sets. This suggests that the misclassified samples collected at 72 h post challenge might suffer from the subjective nature of self-symptom reporting rather than inaccurate prediction.

The 19 biomarker panel was identified using whole blood samples from H1N1 infected adult volunteers which raises the question of generalizability to other influenza strains and cohorts. Zaas et al. [9] developed a 48-gene marker panel to classify H1N1 and H3N2 infected patients as either symptomatic or asymptomatic. Apart from 5 genes (CCL8, CD36, CXCL10, USP18 and SPATS2L), all 14 genes of the 19 gene signature were also found in their panel indicating the potential use of our panel across different strains. Similarly, 12 of our genes also appear in a published signature to detect respiratory infections such as RSV and rhinovirus [10]. Herberg et al. [28] compared whole blood samples from 19 children hospitalized due to H1N1 infection (age 1.6–7.5 years) with 33 control children. Interestingly, 12 genes in their top 15 were also present in our panel. These overlaps strongly suggest that our panel can also be used to predict symptom scores across different influenza strains and age groups as well as for binary classification of volunteers into asymptomatic and symptomatic individuals.

There is also evidence that our set of biomarkers could have potential cross species application once the primers have been adopted for species differences. For example, Li et al. [29] identified ISG15, our most influential predictor, as highly up-regulated in the lung samples from H1N1 infected swine using microarrays and qRT-PCR validation. Another highly influential gene from our panel CXCL10 was shown to be consistently upregulated in H1N1 infected mice, macaques and in swine [30].

Interestingly, we observed a significant further 18% reduction in RMSE, when we predicted scores based on qRT-PCR trained data compared to microarray data. One reason might be the well-known underestimation of expression changes measured by microarrays compared to qRT-PCR [31] which we also clearly observed in our screen (Additional file 8: Figure S5) or the higher sensitivity of qRT-PCR. However, we cannot directly compare both results due to the different cohorts which were used for testing.

Although a 34% reduction in prediction error compared to the mean we observed for our qRT-PCR based assay is a good result, we observed four samples with discordant prediction (Fig. 4a). The inaccuracies observed, seem to be more pronounced when recorded DSS were low in the range of one to three. This result is not surprising, since minor symptoms are expected to be more subjective compared to strong or multiple symptoms.

Selecting a very stringent threshold at the microarray level was a design choice deliberately made at a very early stage of the study for two reasons. At first, we aimed at a cost effective and small set of PCR primers. Secondly, we sought to restrict the primers to genes which were highly and significantly correlated to our phenotype after adjusting for differences in variables such as age, gender and batch. Alternatively, the choice of the genes included in the final signature can be left to a feature selection method or a regression algorithm penalizing and removing less informative features. This however potentially leads to removal of biologically relevant genes and to overfitting to the training data at hand. When we applied a feature selection method to our dataset, we found good predictive ability with even two genes (ISG15 and CXCL10). This would enable a very cost effective approach to symptom scoring. However it requires further testing using a larger cohort, if such a small set of predictors can be a robust marker for symptom scoring.

## Conclusions

Taken together, we provide here a comparatively small set of genes, which can be used to replace self-reported symptom scores in influenza challenge studies with great accuracy. All primers described here to test expression levels of these genes are commercially available and can be readily used to replace or refine self-symptom reporting in influenza challenge trials. Therefore, these markers can in future challenge studies and possibly refine the panel once tested on larger cohorts.

## Additional files

**Additional file 1: Figure S1.** *A*, Overview of the 16 recorded symptom scores for each patient in the microarray study. *B*, Correlation matrix of all symptoms except ‘Wheezy Chest’. A linear model was analysed as outlined in Fig. 1b using limma, but for every symptom separately instead of DSS. The resulting fold change estimates of all genes were correlated using Pearson’s correlation.

**Additional file 2: Table S1.** Table with per sample meta data.

**Additional file 3: Table S2.** Table with per subject daily symptom scores.

**Additional file 4: Figure S2.** Log2 fold change to DSS versus log2 fold change to Live Viral Shedding Assay from two separate linear models. Genes significantly differentially expressed at an FDR of 5% to Live Viral Shedding Assay and DSS are coloured in red and yellow respectively.

**Additional file 5: Figure S3.** Scaled, mean centred microarray expression data (mean 0, standard deviation 1) versus DSS. Symptom scores are in log units for visual clarity.

**Additional file 6: Figure S4.** Heatmap of change in accuracy (units in percentage change in RMSE) when training on the subset of samples on the x-axis and predicting on the subset of test samples from Woods et al. (y-axis). DSS values were log transformed to improve performance on the very small training sets for this figure only. Lower values mean an increase in accuracy.

**Additional file 7: Table S3.** Table of TaqMan primer IDs used for the Fluidigm 96x96 assay.

**Additional file 8: Figure S5.** Scatterplot comparing log2 fold changes per unit of DSS from Illumina ht12 arrays compared to Fluidigm BioMark HD TaqMan assays of 28 selected genes and 3 control genes. One gene, MS4A4A, was omitted due to a missing sample for comparability. Biomarkers used for machine learning are highlighted in green, housekeepers in red and other genes not passing the feature selection process in blue. The identity is indicated as a grey line and a linear regression fit is shown in blue with a 95% CI in grey.

**Additional file 9: Figure S6.** VSURF feature selection of most predictive features. *A*, A random forest model was trained with only the two features ISG15 and CXCL10 as selected by VSURF. Predicted DSS is shown on the x-axis and observed DSS on the y-axis. *B*, Prediction of Any Symptom (DSS > 0) or No Symptom (DSS = 0) classes using the linear classifier Partial Least Square (PLS). Decision boundaries of the trained model are indicated as well as the samples used for training and testing. *C*, ROC curve indicating the accuracy of the PLS model.

**Additional file 10: Figure S7.** *A*, Predicted DSS using the extended 29 gene signature on Fluidigm as a test dataset versus the observed DSS. *B*, Importance of individual markers within the panel of 29 primers. x-axis shows the increase in RMSE if the respective gene is left out of the model. LVS = Live Viral Shedding Assay.

**Additional file 11: Figure S8.** Prediction of Any Symptom (DSS > 0) or No Symptom (DSS = 0) classes using a random forest model on Fluidigm. *A*, Prediction probabilities are shown on the y-axis and samples with probabilities <0.5 are classified as No Symptom. *B*, Importance of individual markers for the categorized prediction within the panel of 19 primers. x-axis shows the decrease in Gini coefficient if the respective gene is left out of the model. *C*, ROC analysis of the categorized prediction.

## Abbreviations

DSS: daily sum of scores
FDR: false discovery rate
HI: haemagglutination inhibition
qRT-PCR: quantitative reverse transcriptase real time polymerase chain reaction
RMSE: root mean squared error
