# Supplementary figures and images for: Development of an objective gene expression panel as an alternative to self-reported symptom scores in human influenza challenge trials
Source: J Transl Med. 2017 Jun 8;15:134. doi: 10.1186/s12967-017-1235-3 (PMC5465537; doi:10.1186/s12967-017-1235-3)

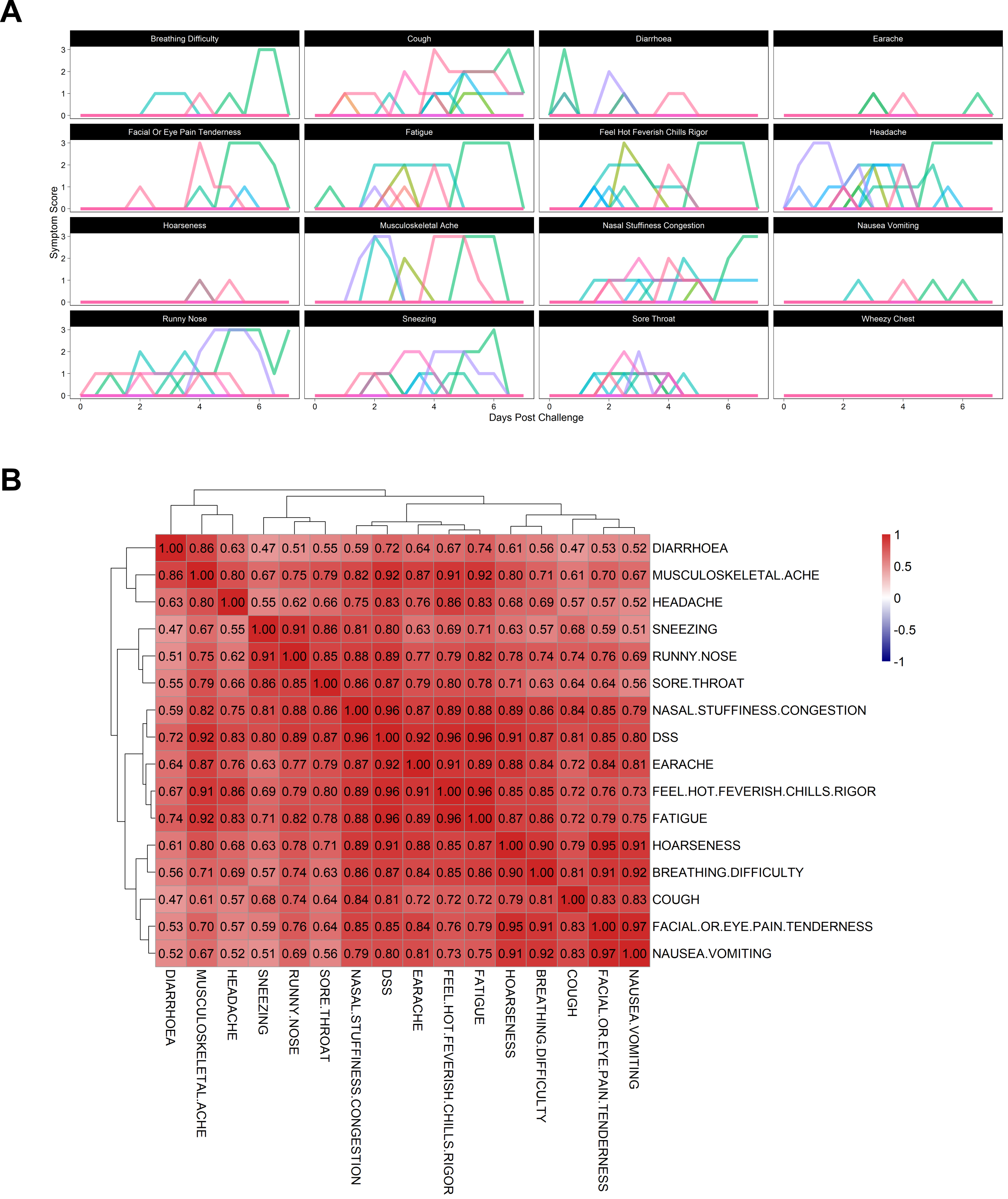

Supplement: Supplementary file 1 — Additional file 1: Figure S1. A, Overview of the 16 recorded symptom scores for each patient in the microarray study. B, Correlation matrix of all symptoms except ‘Wheezy Chest’. A linear model was analysed as outlined in Fig. 1b using limma, but for every symptom separately instead of DSS. The resulting fold change estimates of all genes were correlated using Pearson’s correlation. [file 12967_2017_1235_MOESM1_ESM.png]

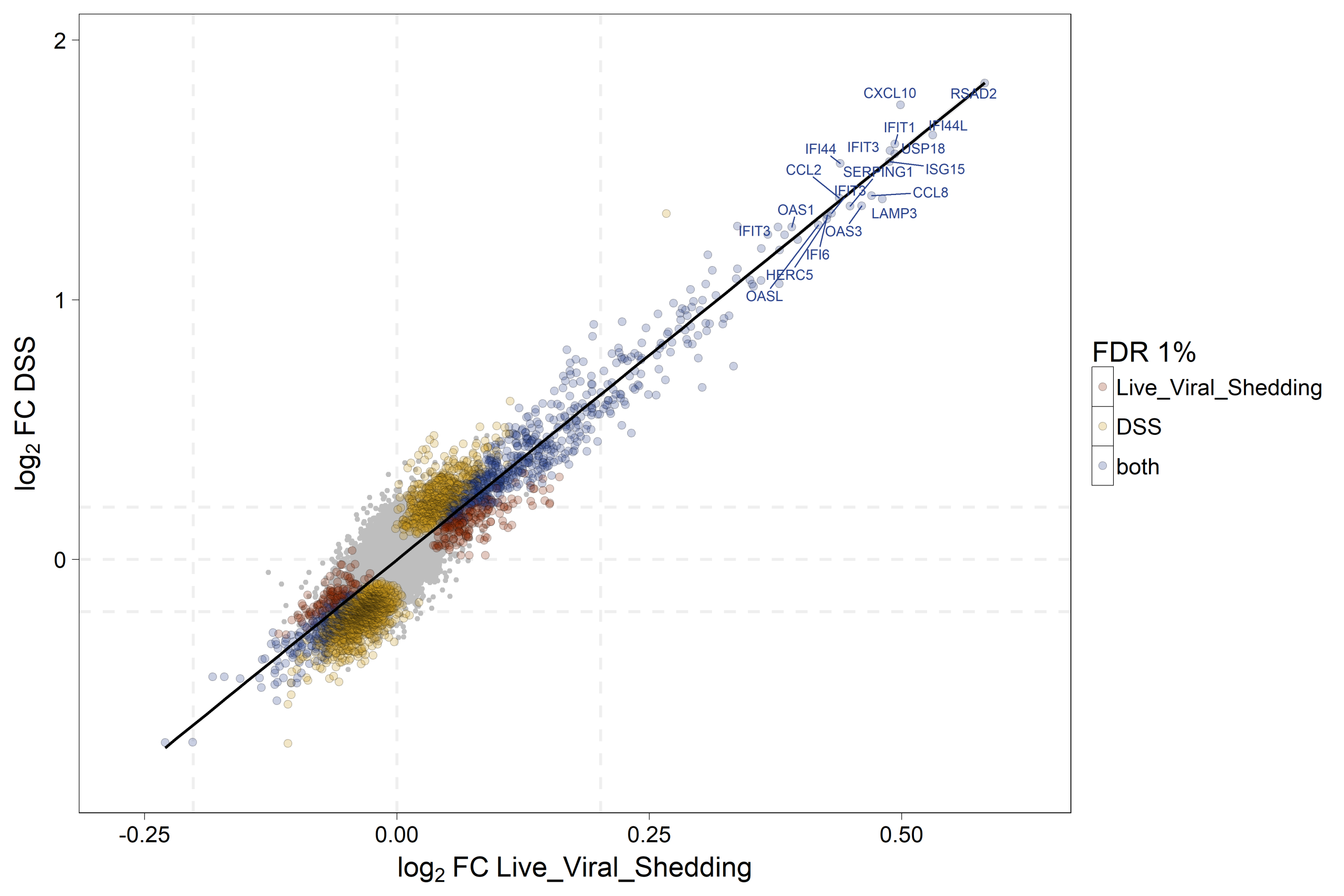

Supplement: Supplementary file 4 — Additional file 4: Figure S2. Log2 fold change to DSS versus log2 fold change to Live Viral Shedding Assay from two separate linear models. Genes significantly differentially expressed at an FDR of 5% to Live Viral Shedding Assay and DSS are coloured in red and yellow respectively. [file 12967_2017_1235_MOESM4_ESM.png]

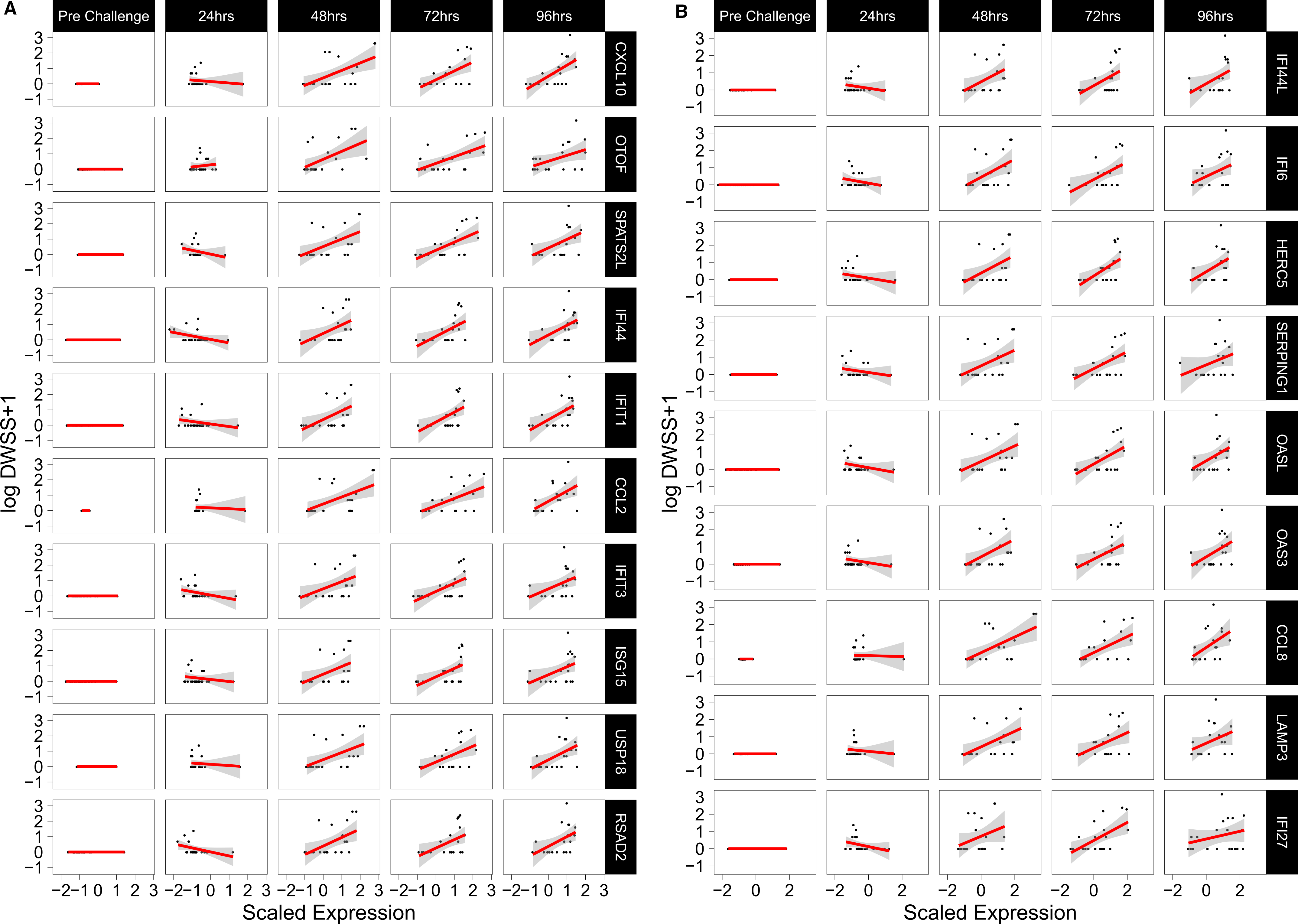

Supplement: Supplementary file 5 — Additional file 5: Figure S3. Scaled, mean centred microarray expression data (mean 0, standard deviation 1) versus DSS. Symptom scores are in log units for visual clarity. [file 12967_2017_1235_MOESM5_ESM.png]

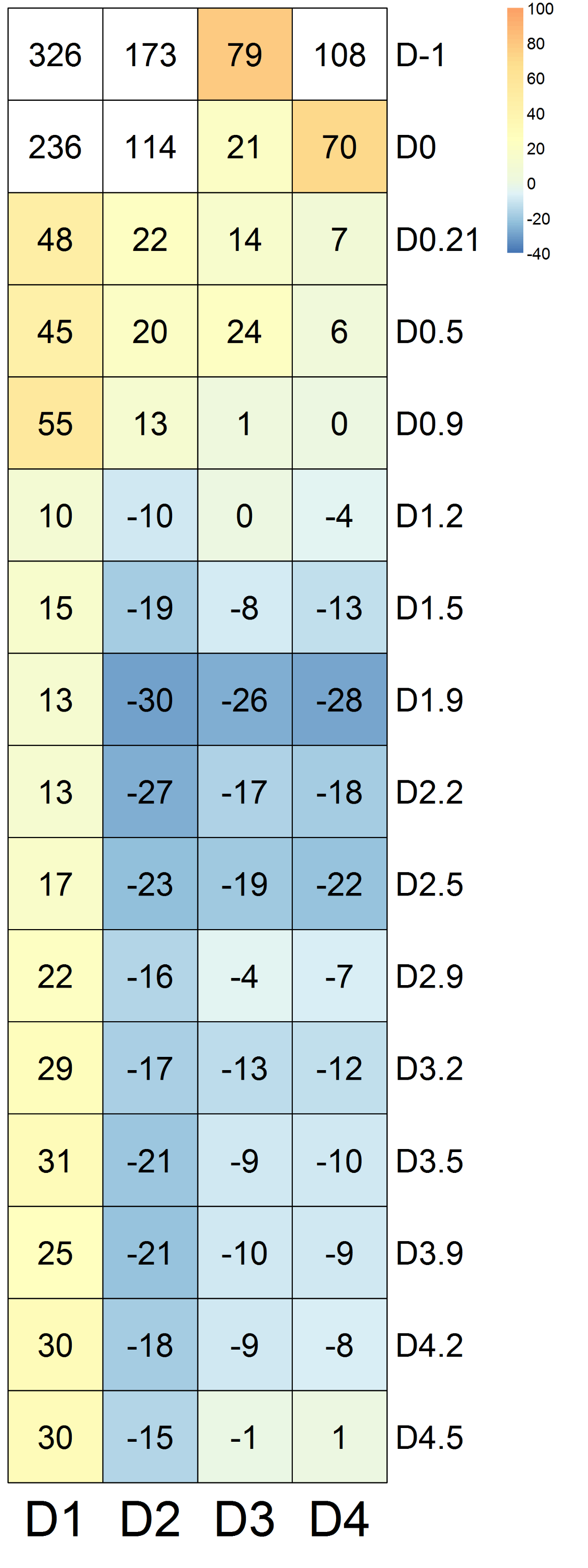

Supplement: Supplementary file 6 — Additional file 6: Figure S4. Heatmap of change in accuracy (units in percentage change in RMSE) when training on the subset of samples on the x-axis and predicting on the subset of test samples from Woods et al. (y-axis). DSS values were log transformed to improve performance on the very small training sets for this figure only. Lower values mean an increase in accuracy. [file 12967_2017_1235_MOESM6_ESM.png]

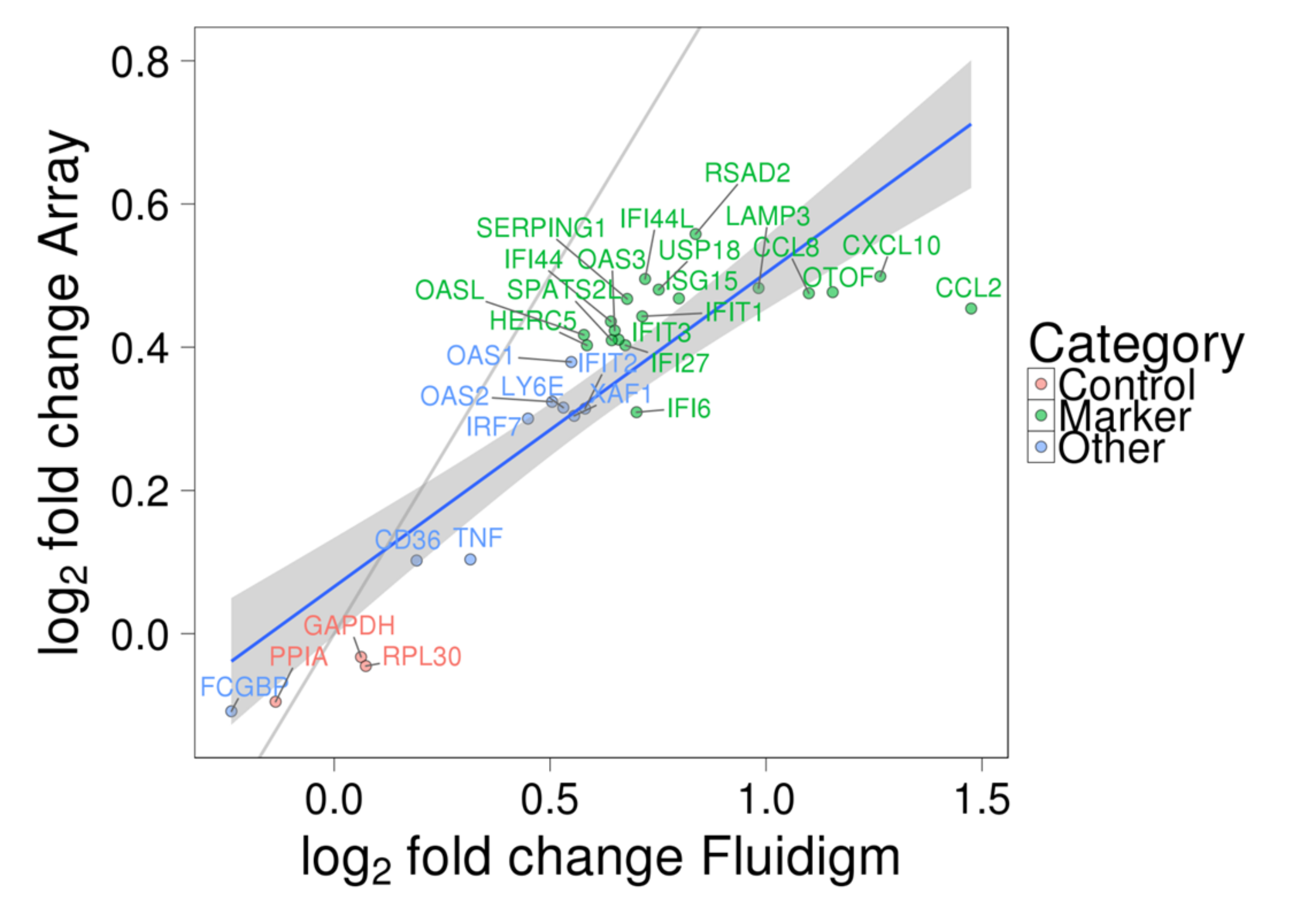

Supplement: Supplementary file 8 — Additional file 8: Figure S5. Scatterplot comparing log2 fold changes per unit of DSS from Illumina ht12 arrays compared to Fluidigm BioMark HD TaqMan assays of 28 selected genes and 3 control genes. One gene, MS4A4A, was omitted due to a missing sample for comparability. Biomarkers used for machine learning are highlighted in green, housekeepers in red and other genes not passing the feature selection process in blue. The identity is indicated as a grey line and a linear regression fit is shown in blue with a 95% CI in grey. [file 12967_2017_1235_MOESM8_ESM.png]

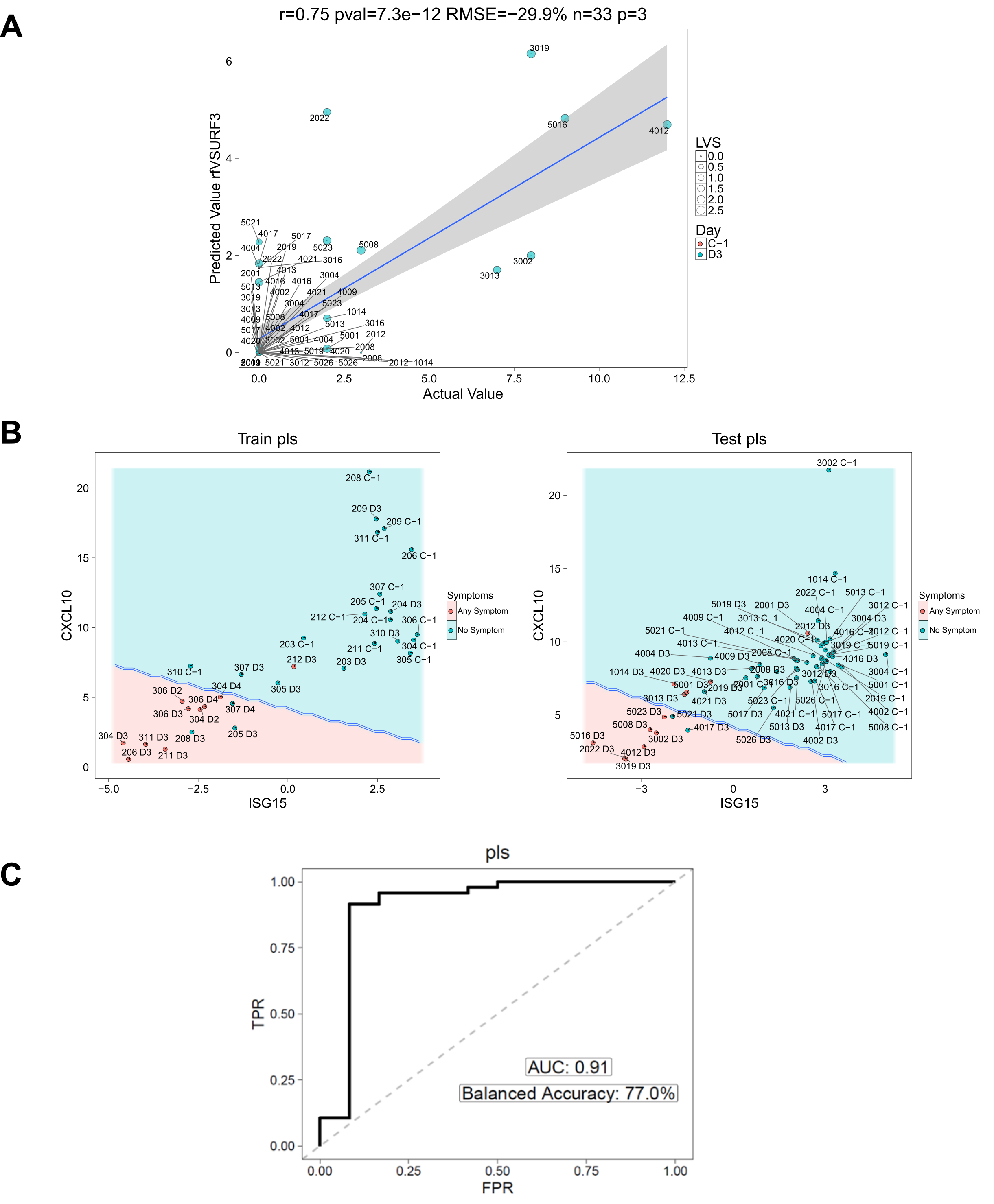

Supplement: Supplementary file 9 — Additional file 9: Figure S6. VSURF feature selection of most predictive features. A, A random forest model was trained with only the two features ISG15 and CXCL10 as selected by VSURF. Predicted DSS is shown on the x-axis and observed DSS on the y-axis. B, Prediction of Any Symptom (DSS > 0) or No Symptom (DSS = 0) classes using the linear classifier Partial Least Square (PLS). Decision boundaries of the trained model are indicated as well as the samples used for training and testing. C, ROC curve indicating the accuracy of the PLS model. [file 12967_2017_1235_MOESM9_ESM.png]

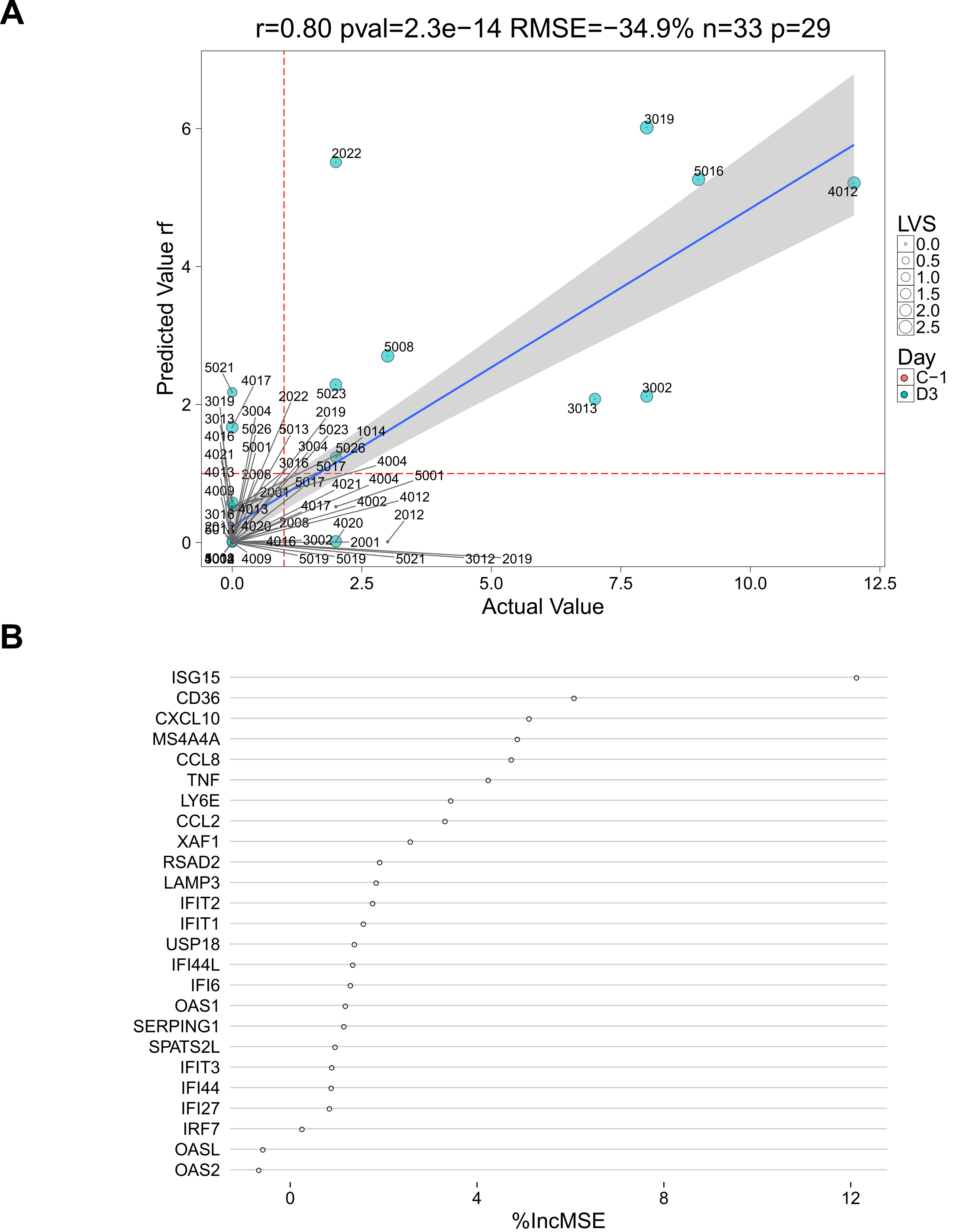

Supplement: Supplementary file 10 — Additional file 10: Figure S7. A, Predicted DSS using the extended 29 gene signature on Fluidigm as a test dataset versus the observed DSS. B, Importance of individual markers within the panel of 29 primers. x-axis shows the increase in RMSE if the respective gene is left out of the model. LVS = Live Viral Shedding Assay. [file 12967_2017_1235_MOESM10_ESM.png]

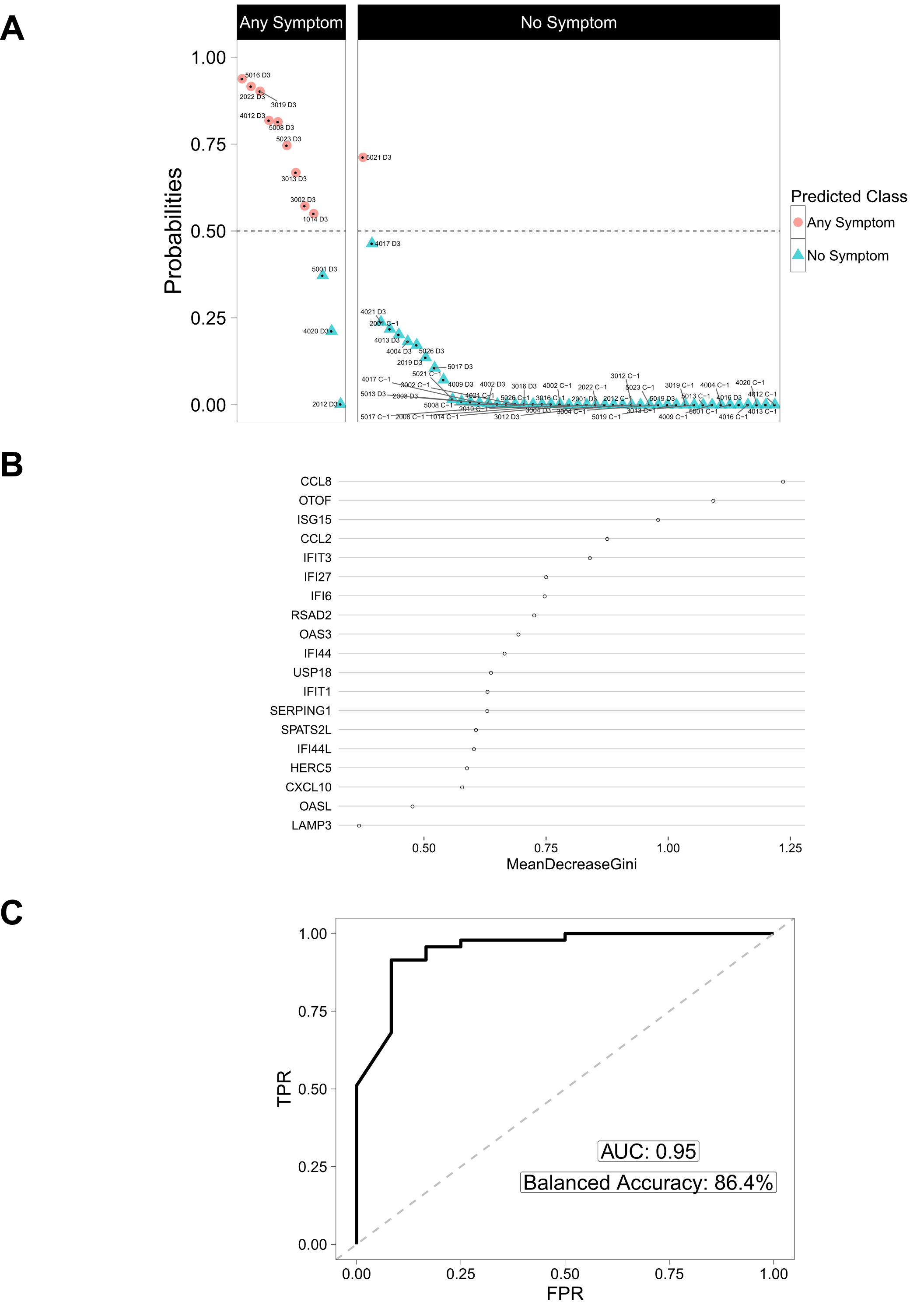

Supplement: Supplementary file 11 — Additional file 11: Figure S8. Prediction of Any Symptom (DSS > 0) or No Symptom (DSS = 0) classes using a random forest model on Fluidigm. A, Prediction probabilities are shown on the y-axis and samples with probabilities <0.5 are classified as No Symptom. B, Importance of individual markers for the categorized prediction within the panel of 19 primers. x-axis shows the decrease in Gini coefficient if the respective gene is left out of the model. C, ROC analysis of the categorized prediction. [file 12967_2017_1235_MOESM11_ESM.png]
