# Supplementary material for: Development of an objective gene expression panel as an alternative to self-reported symptom scores in human influenza challenge trials
Source: J Transl Med. 2017 Jun 8;15:134. doi: 10.1186/s12967-017-1235-3 (PMC5465537; doi:10.1186/s12967-017-1235-3)
Supplement: Supplementary file 7 — Additional file 7: Table S3. Table of TaqMan primer IDs used for the Fluidigm 96x96 assay. [file 12967_2017_1235_MOESM7_ESM.pdf]

Gene	Assay ID	Purpose
CCL2	Hs00234140_m1	DSS
CCL8	Hs04187715_m1	DSS
CXCL10	Hs01124252_g1	DSS
GAPDH	Hs02758991_g1	Control
HERC5	Hs00180943_m1	DSS
IFI27	Hs00271467_m1	DSS
IFI44	Hs00951349_m1	DSS
IFI44L	Hs00915292_m1	DSS
IFI6	Hs00242571_m1	DSS
IFIT1	Hs03027069_s1	DSS
IFIT3	Hs01922752_s1	DSS
ISG15	Hs01921425_s1	DSS
LAMP3	Hs00180880_m1	DSS
OAS3	Hs00196324_m1	DSS
OASL	Hs00984387_m1	DSS
OTOF	Hs00191271_m1	DSS
PPIA	Hs04194521_s1	Control
RPL30	Hs00265497_m1	Control
RSAD2	Hs00369813_m1	DSS
SERPING1	Hs00163781_m1	DSS
SPATS2L	Hs01016364_m1	DSS
USP18	Hs00276441_m1	DSS
